# Machine learning approaches and non-linear processing of extracted components in frontal region to predict rTMS treatment response in major depressive disorder

**DOI:** 10.3389/fnsys.2023.919977

**Published:** 2023-03-09

**Authors:** Elias Ebrahimzadeh, Farahnaz Fayaz, Lila Rajabion, Masoud Seraji, Fatemeh Aflaki, Ahmad Hammoud, Zahra Taghizadeh, Mostafa Asgarinejad, Hamid Soltanian-Zadeh

**Affiliations:** ^1^School of Electrical and Computer Engineering, College of Engineering, University of Tehran, Tehran, Iran; ^2^School of Cognitive Sciences, Institute for Research in Fundamental Sciences (IPM), Tehran, Iran; ^3^Biomedical Engineering Department, School of Electrical Engineering, Payame Noor University of North Tehran, Tehran, Iran; ^4^School of Graduate Studies, SUNY Empire State College, Manhattan, NY, United States; ^5^Department of Psychology, University of Texas at Austin, Austin, TX, United States; ^6^Department of Biomedical Engineering, Islamic Azad University Central Tehran Branch, Tehran, Iran; ^7^Department of Medical and Technical Information Technology, Bauman Moscow State Technical University, Moscow, Russia; ^8^Department of Bioengineering, George Mason University, Fairfax, VA, United States; ^9^Department of Cognitive Neuroscience, Institute for Cognitive Sciences Studies, Tehran, Iran

**Keywords:** electroencephalography (EEG), repetitive transcranial magnetic stimulation (rTMS), major depressive disorder (MDD), prediction treatment response, independent component analysis (ICA), classification, machine learning approaches, non-linear processing

## Abstract

Predicting the therapeutic result of repetitive transcranial magnetic stimulation (rTMS) treatment could save time and costs as ineffective treatment can be avoided. To this end, we presented a machine-learning-based strategy for classifying patients with major depression disorder (MDD) into responders (R) and nonresponders (NR) to rTMS treatment. Resting state EEG data were recorded using 32 electrodes from 88 MDD patients before treatment. Then, patients underwent 7 weeks of rTMS, and 46 of them responded to treatment. By applying Independent Component Analysis (ICA) on EEG, we identified the relevant brain sources as possible indicators of neural activity in the dorsolateral prefrontal cortex (DLPFC). This was served through estimating the generators of activity in the sensor domain. Subsequently, we added physiological information and placed certain terms and conditions to offer a far more realistic estimation than the classic EEG. Ultimately, those components mapped in accordance with the region of the DLPFC in the sensor domain were chosen. Features extracted from the relevant ICs time series included permutation entropy (PE), fractal dimension (FD), Lempel-Ziv Complexity (LZC), power spectral density, correlation dimension (CD), features based on bispectrum, frontal and prefrontal cordance, and a combination of them. The most relevant features were selected by a Genetic Algorithm (GA). For classifying two groups of R and NR, K-Nearest Neighbor (KNN), Support Vector Machine (SVM), and Multilayer Perceptron (MLP) were applied to predict rTMS treatment response. To evaluate the performance of classifiers, a 10-fold cross-validation method was employed. A statistical test was used to assess the capability of features in differentiating R and NR for further research. EEG characteristics that can predict rTMS treatment response were discovered. The strongest discriminative indicators were EEG beta power, the sum of bispectrum diagonal elements in delta and beta bands, and CD. The Combined feature vector classified R and NR with a high performance of 94.31% accuracy, 92.85% specificity, 95.65% sensitivity, and 92.85% precision using SVM. This result indicates that our proposed method with power and nonlinear and bispectral features from relevant ICs time-series can predict the treatment outcome of rTMS for MDD patients only by one session pretreatment EEG recording. The obtained results show that the proposed method outperforms previous methods.

## 1. Introduction

Major Depressive Disorder (MDD), more commonly known as clinical depression, is a severe condition with potential morbidity and mortality, affecting and threatening millions of people worldwide. MDD is typically treated with one type of antidepressant, however, 50% to 70% of patients are shown to be categorically unresponsive to medication-based treatments (Chekroud et al., 2017; Ebrahimzadeh et al., 2021a; Leichsenring et al., 2022; Turner et al., 2022). Therefore, there has been an ongoing search for other therapeutic approaches to target patients with resistant depression. One method that has gained increasing attention as a safe alternative or complementary technique to treat MDD is repetitive Transcranial Magnetic Stimulation (rTMS; Čukić, 2020). rTMS involves a series of short magnetic pulses directed to the brain to stimulate nerve cells. The magnetic pulses stimulate area neurons and change the functioning of the brain circuits involved. This method is a noninvasive treatment that directs magnetic pulses at the left or right dorsolateral prefrontal cortex (DLPFC) at regular intervals to stimulate neurons and trigger action potentials. It may be used as an adjunctive therapy to increase or hasten the efficacy of conventional pharmacotherapy through changing and modulating cortical activity. The overall effectiveness and limited side effects of rTMS have been established in several studies. Nonetheless, clinicians prescribe rTMS after conducting a thorough assessment and a series of trial-and-error tests to improve diagnostic accuracy and treatment outcomes and, importantly, to prevent patient relapse which may be the case if the patient is unresponsive to rTMS. This calls for developing indicators that can help with predicting rTMS response in order for patients to benefit from the merits of this treatment and avoid costly, ineffective procedures. Neurophysiological modalities, including fMRI and EEG, have been used to this end, between which, EEG, being more widely available and cost-effective, makes a more robust biomarker (Bachmann et al., 2013; Patel et al., 2015; Redlich et al., 2016; Wade et al., 2016; Čukić et al., 2020). This is why an increasing number of studies have applied EEG-based machine learning techniques and statistical methods to distinguish rTMS responders from non-responders (Bares et al., 2007; O’Reardon et al., 2007; Khodayari-Rostamabad et al., 2011; Arns et al., 2012, 2014; Kito et al., 2012).

The literature also includes similar efforts to determine the responsiveness of other approaches to resistant MDD treatment. A number of studies, such as Bares et al. (2007) have dealt with regarding changes in QEEG prefrontal cordance as a predictor of response to antidepressants. The effectiveness of selective serotonin reuptake inhibitors (SSRI), as another potential predictor of treatment response, has been investigated in the study of Khodayari-Rostamabad et al. (2013), where Khodayari-Rostamabad et al. obtained the patient’s initial EEG and used it to extract features and perform a mixture of factor analysis (MFA) model. This led to a classification accuracy of 87.9%. SSRI efficacy was also examined in another study, where logistic regression (LR) was applied to wavelet features of baseline EEG, producing an accuracy of 87.5% (Mumtaz et al., 2017). Transcranial Direct-Current Stimulation (tDCS) is another treatment for mood and cognition improvement in patients with MDD, which was the focus of Al-Kaysi et al. (2017). The authors made use of Linear Support Vector Machine (LSVM), Linear Discriminant Analysis (LDA), and neural networks to classify features extracted from EEG and achieved an accuracy of 76% and 92% in mood and cognition labeling, respectively.

As for rTMS, the authors (Cao et al., 2018a) provided a meta-analysis reporting relatively low rates of 40.9% and 16.4% for response and remission, respectively. Bailey et al. (2018) examined EEG recordings when participants were completing a Working Memory (WM) task and predicted rTMS response with an accuracy of 91%. In another effort, they obtained resting state EEG at baseline and 1 week after the start of the treatment and combined mood and EEG features using an LSVM classifier. This yielded a classification accuracy of 86.6% (Bailey et al., 2019). The extracted EEG features they used included EEG power and weighted phase lag index (wPLI) in alpha and theta (Bailey et al., 2019) and gamma (Bailey et al., 2018) frequency bands, alpha peak frequency (iAPF), and frontal theta cordance (Bailey et al., 2019; Jaworska et al., 2019). The literature also includes other EEG features used for the same purpose such as power spectral features (Khodayari-Rostamabad et al., 2013; Al-Kaysi et al., 2017), coherence (Khodayari-Rostamabad et al., 2013; Mumtaz et al., 2017), mutual information (MI; Khodayari-Rostamabad et al., 2013), nonlinear features (Hasanzadeh et al., 2019), time-frequency processing (Ebrahimzadeh et al., 2021a), and wavelet coefficients (Mumtaz et al., 2017). Among these, EEG power in different frequency bands and their combinations have received a lot of attention in predicting MDD treatment (Suffin and Emory, 1995; Knott et al., 2000; Cook et al., 2002; Bruder et al., 2008; Spronk et al., 2011; Tenke et al., 2011; Arns et al., 2012; Pellicciari et al., 2013; Wade et al., 2016; Lebiecka et al., 2018). For instance, treatment response was shown to be associated with cordance measures (Leuchter et al., 1994) and, in another study, with an Antidepressant Treatment Response (ATR) index (Iosifescu et al., 2009). Olejarczyk et al. (2020) evaluated the impact of rTMS on functional connectivity in MDD and bipolar disorder by directed transfer function and indices based on graph theory.

A number of studies have brought their attention to the non-Gaussian, higher-order nature of EEG to discover supplementary information that is not detected in the power spectrum. Adopting this view, Hasanzadeh et al. (2019) used non-linear and bispectral features to classify rTMS response.

Furthermore, a large strand of literature employs three prefrontal electrodes, i.e., FP1, FP2, and FPz, to extract predictive features, as the frontal lobe is believed to contain significant changes in MDD. Such studies tend to limit their analyses to the outcome of those electrodes, which could be interpreted as a simplification of the matter: there is little guarantee that the frontal components would not affect the channels from other areas namely the central, parietal, temporal, and occipital. It can then be stated that the components from the frontal lobe are more involved than those from other areas. Identifying these components and extracting features from their time series could lead to more realistic results compared to those of the other EEG channels. In other words, the frontal components form a neural network, which is involved in the rTMS treatment, and the EEG channels reflect their activity. That said, this article tries to shift the focus to the component domain. To do this, we first had the channels decomposed to their components and identified the appropriate components through analyzing their locations and the dipoles related to the MNI model. We then used the time series of the selected components to extract features and performed the classification. To choose predictive features for rTMS treatment response, we investigated a relatively comprehensive set of component’s time-series features including spectral, bispectral, and nonlinear features, namely bispectrum features, Lempel-Ziv Complexity (LZC), correlation dimension (CD), fractal dimension (FD), component power in all frequency bands (delta, theta, alpha, and beta), and frontal and prefrontal cordance in the theta band, all extracted from pretreatment resting EEG. In addition to classification, we performed a statistical test to evaluate the differences of features in two groups of responders (R) and non-responders (NR). Ultimately, we employed a Genetic Algorithm (GA) for feature selection. To the extent of our knowledge, this is the first time that the capability of selected bispectral, nonlinear, and spectral features on the components time-series have been investigated simultaneously with the aim of treatment response prediction.

**Figure 1:**
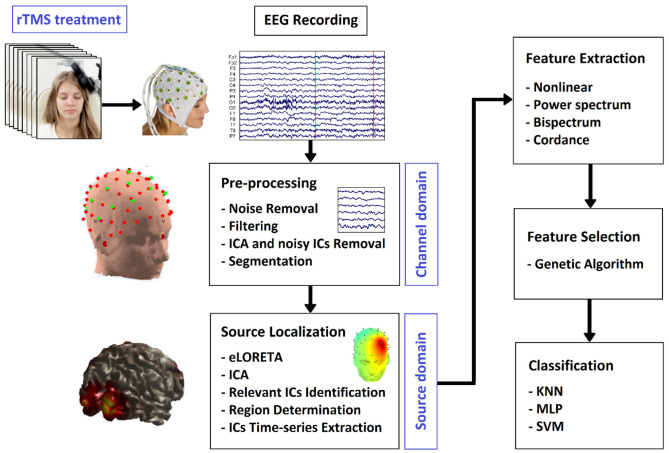
Block diagram of the proposed approach for prediction of rTMS treatment response in MDD.

As illustrated in Figure 1, we first provide the information of participants, and the procedure of EEG acquisition. After extracting the relative components and features, we perform the classification and statistical test. Based on our classification study, we will evaluate the prediction ability of feature sets in the Results Section. The features will then be used to categorize the subjects into two groups of R and NR based on our statistical analysis. The results are elaborated in the Discussion Section where the limitations of the current study and suggestions for future work are also presented. The article is concluded in the Conclusion section.

## 2. Methods and material

### 2.1. Participants

We recruited 88 patients with MDD in the age range of 18–54 years. The patients were referred to the Neuraly Clinical Neuroscience Centre, Tehran, Iran. A psychiatrist diagnosed them with MDD using the Diagnostic and Statistical Manual-IV (DSM-IV) diagnostic criteria (Segal, 2010). The Hamilton Rating Scale for Depression (HRSD) and the Beck Depression Inventory were also used to assess the participants (BDI-II). All participants have an HRSD score ≥12 and a BDI-II score ≥15. The demographic and clinical data of the participants are described in Table 1. Wilcoxon rank-sum and Friedman tests were used to compare the R and NR groups, with the results displayed in column 4 of Table 1.

**Table 1:** Clinical and demographic information.

	**Responder (*n* = 46)**	**Non-Responder (*n* = 42)**	**Statistics**
Sex (F/M)	27/19	25/17	*P* = 0.940
Age (SD)	41.2 (15.5)	40.3 (13.8)	*P* = 0.108
Years of education (SD)	14.2 (2.3)	14.1 (2.6)	*P* = 0.375
Handedness (R/L/A)	22/18/6	26/15/1	*P* = 0.101
HDRS	24.5 (4.3)	23.3 (4.4)	*P* = 0.921
Treatment (HF/LF/bilateral)	14/17/15	11/13/18	*P* = 0.481
Number of previous medications	2.8 (1.5)	2.2 (1.8)	*P* = 0.214
Medications (AD/ AD ± MS / AD ± MS ± AP)	16/21/9	19/21/2	*P* = 0.592
Anxiety (Y/N)	34/12	27/15	*P* = 0.131
Disease duration (years)	5.3 (7.6)	6.4 (8.3)	*P* = 0.268
Pre-treatment BDI-II	31.2 (10.1)	29 (9.6)	*P* = 0.098
Post-treatment BDI-II	9 (4.2)	23 (7.4)	*P* < 0.001
Pre-treatment HRSD	36 (7.3)	28 (3.1)	*P* = 0.193
Post-treatment HRSD	7 (5.4)	26 (6.5)	*P* < 0.001

M, Male; F, Female; SD, Standard Deviation; L, Left; R, Right; A, Ambidextrous; HDRS, 17-item Hamilton Depression Rating Scale. HF, High Frequency (10 Hz); LF, Low Frequency (1 Hz); AD, Antidepressant; MS, Mood Stabilizer; AP, Antipsychotic.

In terms of age, gender, rTMS treatment features, pretreatment BDI-II and HRSD score, medications, duration of illness, length of current depression episode, and the number of previous drugs, there were no significant differences (*p* > 0.05) between R and NR. Only the posttreatment BDI-II and 17-item HRSD scores differed significantly (*p* < 0.05), with responders scoring significantly lower. The outcome of rTMS cannot be attributed to differences in depression severity of the two groups because the pretreatment BDI-II and HRSD of R and NR are not significantly different. The existence of Axis I or II disorders, substance misuse, suicide risk, unstable medical conditions, implanting devices, cardiac arrhythmia, and pregnancy are all exclusion criteria in this study. Participants having a current or previous head injury, seizures, epilepsy, or neurological diseases were also excluded from the study. In this study, 59 individuals had been on antidepressants, mood stabilizers, and antipsychotics for more than 4 weeks prior to the treatment. The Bioethics Committee of the Iran University of Medical Science authorized all experimental techniques, which followed the principles of the Declaration of Helsinki standards. All participants volunteered and provided written consent after being told about the study procedures and goals. Table 1 summarizes the demographic and clinical information of the participants.

### 2.2. Procedure and clinical assessment

A baseline interview was conducted with MDD patients to collect demographic and depression severity data. The 17-item Hamilton Rating Scale for Depression (HRSD; Sharp, 2015), the Montgomery-Asberg Depression Rating Scale (MADRS; Leentjens et al., 2000), and the Beck Depression Inventory-II (BDI-II; Dozois et al., 1998) were used to determine the severity of depression.

**Figure 2:**
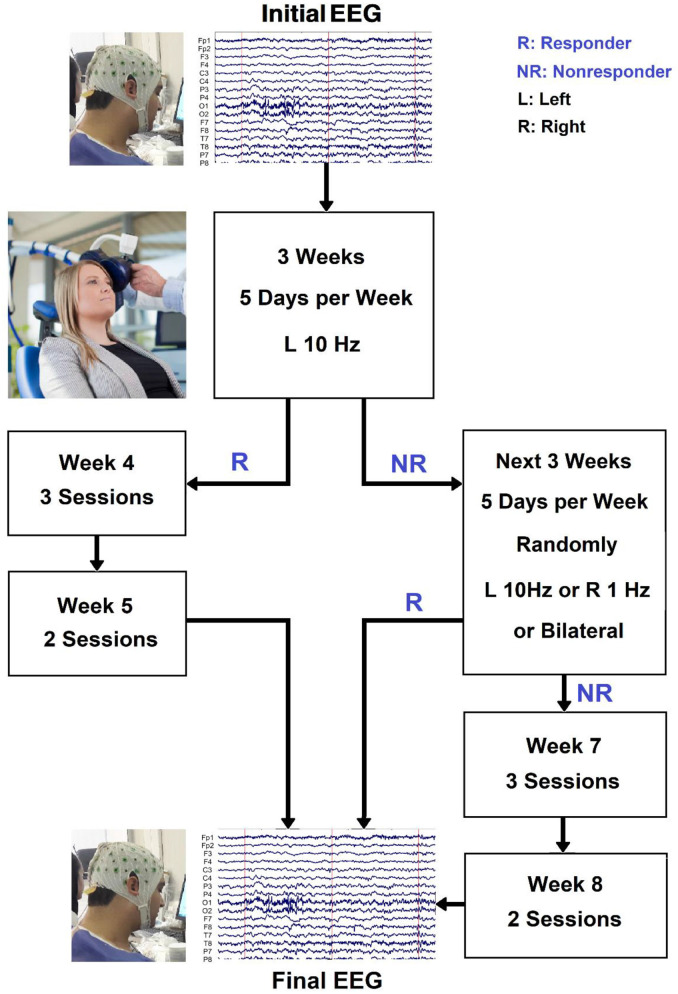
The procedure for rTMS treatment and the selection of two responsive (R) and nonresponsive (NR) groups.

MDD patients received daily (5 days per week) unilateral left 10 Hz rTMS therapy for 3 weeks. Individuals who responded after 3 weeks were given an extra 2 weeks of titrated rTMS treatment (three sessions in week 4, two sessions in week 5), followed by another EEG session. Nonresponders were randomly assigned to continue with unilateral left 10 Hz rTMS therapy, unilateral right 1 Hz rTMS treatment, or sequential bilateral rTMS treatment consisting of right 1 Hz rTMS followed by left 10 Hz rTMS treatment for the next 3 weeks. Individuals who did not respond by week 3 but did by week 6 were given another 2 weeks of titrated rTMS treatment (three sessions in week 7, two sessions in week 8). The procedure for rTMS treatment is shown in Figure 2. The stimulation intensity was set at 110% of the resting motor threshold. The left-sided treatment consisted of 40 5-s trains separated by a 25-s break. One train of 1,200 pulses was used for right-sided rTMS. These procedures were combined in bilateral rTMS, but with only 900 right-sided pulses. The coil was held tangentially to the head and its handle facing back and away from the midline at 45°, and the rTMS was applied to the left/right DLPFC and bilateral DLPFC at a point 5 cm anterior in a parasagittal line to the motor threshold location (the left abductor pollicis brevis muscle). A TAMAS (REMED, Daejeon, South Korea) with a figure-of-eight-shaped coil (field strength 3 Tesla) delivered the rTMS. At the end of week 1 and week 3, the MADRS and BDI-II tests were redone. Individuals who had not responded by week 3 but had responded by week 6 were assessed using the MADRS, BDI-II, and HRSD at weeks 6 and 8. Anxiolytics and hypnotics were allowed at stable levels before the start of the rTMS therapy, but not 8 h before an EEG recording. During the study period and for at least 5 days before EEG recording and the first rTMS session, no antidepressants, antipsychotics, or anticonvulsants were allowed. It is necessary to mention since it can be quite a serious ethical and medical issue to prohibit the use of antidepressants for patients with depression, whose pre-treatment HRSD scores indicate severity of about 30 points, individuals who had these conditions were excluded from participating in this study. At the beginning and end of the treatment, resting EEG was obtained. The participants’ EEGs were recorded for 5 min in each session while they sat in a comfortable chair in a shielded room with their eyes closed (Ahmadlou et al., 2012; Hasanzadeh et al., 2019). The participants were told not to fall asleep during the experiment. Responding to treatment is defined as more than 50% decrease in BDI_II scores or HRSD or by BDI ≤ 8 (HRSD ≤ 7) which indicates remission (Hasanzadeh et al., 2019).

### 2.3. EEG recording and pre-processing

All EEG recordings took place at the Neuraly Clinical Neuroscience Centre, Tehran, Iran. A 32-channel eWave32 amplifier was used for EEG signal recording which followed the International 10–20 System of electrode placement on the scalp. The amplifier, made by ScienceBeam^1^, has a sampling rate of 1k samples/second, allowing us to precisely study the temporal dynamics of information processing in the brain (with a referential montage, where the reference electrode was placed in the FCz position; Raeisi et al., 2020; Seraji et al., 2021; Seraji, 2021). We applied standard pre-processing procedures in EEGLAB (available at https://sccn.ucsd.edu/eeglab/ version 2021) to reduce noise and artifacts from the EEG signals (Ebrahimzadeh et al., 2021b). First, the sampling rate of the signal was reduced to 250 Hz, and filtered by a Butterworth band-pass filter at 1–60 Hz. Then, all the channels were reviewed, and those with a standard deviation greater than ±3.1 from the mean standard deviation (across all channels) were excluded as the channels that contain artifact. For eliminating the power-line noise at 50 Hz, the Clean Line algorithm was used (Ebrahimzadeh et al., 2019a,b). The advantage of this algorithm over the notch filter is that it adaptively estimates and removes sinusoidal artifacts without creating band-holes in the EEG power spectrum (Ebrahimzadeh et al., 2021b). Next, the ICA algorithm was applied on the EEG signal and the irrelevant components corresponding to eye blink, eye movement, cardiac pulsatile, muscular tension, swallowing, or machine vibration were visually identified using the component’s scalp map, spectral power activity, and spectral power distribution (Ebrahimzadeh et al., 2019c, 2021c; Sadjadi et al., 2021). After identifying all the artifact components, the data were re-composed without them. Finally, the average reference was used to re-reference all of the data. We kept 300 s of each subject’s EEG signal to equalize the length of data for all participants, taking into account the parts of the EEG data that were eliminated.

### 2.4. LORETA

To determine brain electrical sources, researchers use the LORETA method (low resolution electromagnetic tomography). LORETA is a Laplacian-weighted minimum norm algorithm that relies on the patient’s prior neuroanatomical and physiological knowledge as well as a mathematical restriction. The method is based on projecting the brain’s electric activity onto all of the points in a 3D grid. Unlike dipole source modeling approaches, every site whose activity is reformed is considered a potential source (Jaworska et al., 2019). As a result, the model does not require a predetermined number of sources. The smoothest spatial distribution is chosen by minimizing the Laplacian of weighted current sources. The idea is that neighboring voxels should have an electrical activity that is as comparable as possible, such as the same orientation and activation. The LORETA approach, being time dependent, allows for an inverse solution by using spatial coefficients as input. It is sufficient to have a one-time sample to generate a combination of sources. That said, we separated EEG activities into time windows for a solution, and investigated the 5-s segments.

### 2.5. Independent component analysis (ICA)

**Figure 3:**
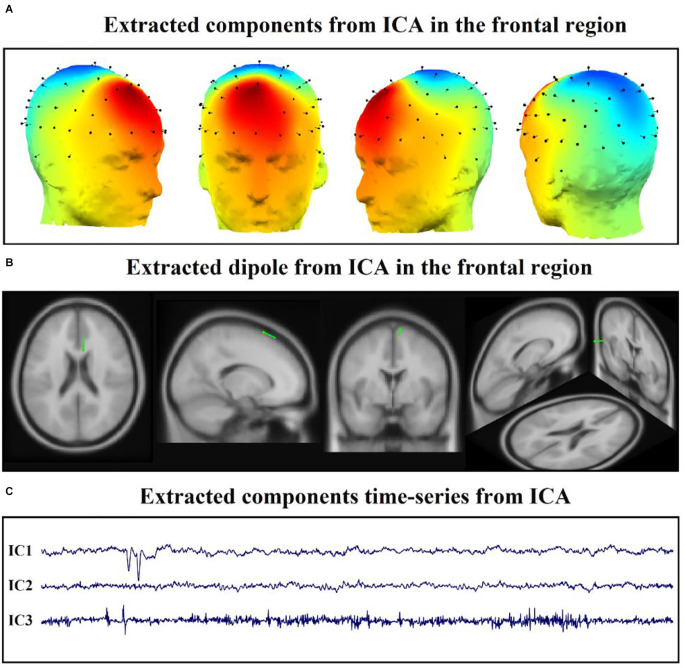
Application of ICA on EEG after rTMS treatment. **(A)** Extracted components from ICA in the frontal region. **(B)** Extracted dipole from ICA in the frontal region. **(C)** Extracted components time-series from ICA in the mentioned region.

Independent Component Analysis (ICA) is used for a statistical decomposition of multi-channel EEG signals for source separation. The EEG signal is made up of a variety of contributions, and utilizing this method, independent components can be isolated from the mixed signals. ICA converts a multivariate random signal into a signal with mutually independent components. We extracted the time series of each component of the DLPFC area and then elicited the features. We chose the first three components to study since each participant had at least three components in the DLPFC region, and the first three components are more likely to play a substantial role (Figure 3; Ebrahimzadeh et al., 2019a,c, 2021b,c).

#### 2.5.1. Region of interest (ROI)

Referring to the existing literature, we have defined a region-of-interest method to obtain the volumetric measurements of DLPFC. In a number of studies, it is shown that the DLPFC site is optimally identified as the midpoint of a line drawn between the F3 and AF3 EEG points (Fitzgerald et al., 2009; Ahdab et al., 2010; Peleman et al., 2010). In addition, authors of Paxinos and Mai (2015), Brodmann (1909), Fischl and Dale (2000), and von Economo et al. (2008) have highlighted the fact that the human cerebral cortex is a highly folded sheet of neurons with a thickness varying from 1–4.5 mm (overall average being around 2.5 mm). Considering both views, we have determined the ROI as a cylinder that has a circle centered in the middle of F3 and AF3 with a radius of 25 mm and a thickness of 2.5 mm.

### 2.6. Feature extraction

After extracting the time series of linked components, features extraction is the next stage in predicting treatment response to rTMS. We evaluated a total of 23 features in four categories: nonlinear, spectral, bispectral, and cordance. Each feature (excluding cordance) was computed for all selected components of both groups of R and NR. Thus, for each measure, we have a feature set containing 23 feature vectors corresponding to three components making a total of 69 features. The next sections describe the measures that were investigated.

#### 2.6.1. Power spectrum

The signal’s power in its frequency components is represented by the power spectrum. Delta (1–4 Hz), Theta (4–8 Hz), Alpha (8–12 Hz), and Beta (12–24 Hz) are common frequency bands. The components time series were used to calculate the average powers. The power spectrum of the components bands was calculated using a Fast Fourier Transform based on the Welch method for each band of each component. The Welch’s approach separates the signal of N samples into K data segments of M samples, with D samples possibly overlapping. After that, the overlapping portions are windowed, i.e., multiplied by a symmetric bell-shaped window. The periodogram of each windowed data segment can then be computed using the discrete Fourier transform (DFT). The average adjusted periodogram of all segments is then calculated as the final estimate of the spectrum. In this study, a non-overlapped window with 1,000 samples (1 s length) was chosen for each band of each component.

#### 2.6.2. Cordance

Cordance is a quantitative EEG approach that embodies information from the EEG spectra’s absolute and relative powers. Many researchers have used cordance as a way of distinguishing depression and predicting treatment outcomes (Bares et al., 2015; Baskaran, 2016; Bailey et al., 2018, 2019). Age, gender, and the degree of baseline depression have little impact on cordance. The ratio of the delta, theta, alpha, or beta band power over the total power in the full frequency range, which eliminates individual differences, has been termed as the relative EEG power. Some of the previous studies have shown decreased prefrontal cordance of the theta frequency band after the treatment (Cook et al., 2002; Bares et al., 2007, 2015; Cao et al., 2018b; Bailey et al., 2019). Therefore, in MDD, prefrontal theta cordance can be a documented neurophysiological biomarker for predicting response to antidepressants. In this work, theta cordance from extracted component time series in prefrontal was calculated according to the following three steps:

First, the power spectra (*P*) were averaged for each component. The absolute and the relative power (*P*_*s,f*_ and ${\bar{P}}_{s,f}$ respectively) were expressed as:


(1) $${\bar{P}}_{s,f}=\frac{P_{s,f}}{P_{s,f^{\prime }}}$$

where *s* is the electrode site and *f* and *f’* are the specific frequency band and all bands, respectively.

Second, the absolute and relative EEG powers were normalized by dividing them by the maximum absolute and relative powers in each frequency band (f) as:


(2) $$P_{ANORM(s,f)}=\frac{P_{\left(s,f\right)}}{\max\left(P_{s}\right)}$$

(3) $$P_{RNORM(s,f)}=\frac{{\bar{P}}_{\left(s,f\right)}}{\max\left({\bar{P}}_{s}\right)}$$

Finally, the cordance values at each electrode site (*s*) for each frequency band (*f*), *C*_*s,f*_, were calculated as:


(4) $$C_{s,f}=(P_{ANORM(s,f)}-0.5)+(P_{RNORM(s,f)}-0.5)$$

In this study, we calculated theta cordance from extracted component time series in prefrontal.

#### 2.6.3. Nonlinear features

Various methods have been proposed over the last two decades for obtaining EEG nonlinear properties to characterize brain activities. The first and most important characteristic of EEG is its dynamic “complexity,” which may be quantified *via* complexity analysis. The degree of unpredictability in time series is mostly represented by the complexity analysis. In this study, we computed four nonlinear features. All nonlinear measures were calculated for epochs of component time series with the length of 3,000 samples and then have been averaged over all epochs. Thus, for every component, we have four values that are corresponding to each nonlinear measure for every subject.

##### 2.6.3.1. Permutation entropy (PE)

Permutation entropy (PE) is a new feature extraction method, which has low computational complexity, robustness, and simplicity (Grova et al., 2008; Berger et al., 2017; Ebrahimzadeh et al., 2018a; Hasanzadeh et al., 2019; Čukić et al., 2020). It can be used to investigate the local order structure of a dynamic time series and measure the degree of regularity in the EEG data. PE turns an EEG time series into an ordinal pattern sequence. It converts non-stationary time series into a set of ordinal patterns, each of which describes the order relationship between the present and a set of equidistant previous values at a given point in time.

The PE values reported in this work were divided by log(m!) for normalization. They are dimensionless quantities in the interval [0, 1] (Berger et al., 2017).

For a scalar time series X(n) = [x (1), x (2),‥., x (n)], the reconstruction time series is:


(5) X(i) = [x(i), x(i+τ), ..., x(i+(m+1)τ)] I = 1, 2, ... , n (m−1)τ

where *m* is “the length of the pattern”, that is the number of sample points included in each pattern and the “time lag” (τ) is the number of samples that spanned each section of the pattern. Then, x(i) is rearranged in increasing order:


(6) $$\text{x}\left(\text{i}+\left({\text{j}}_{1}-1\right)\tau \right)\leq \text{x}\left(\text{i}+\left({\text{j}}_{2}-1\right)\tau \right)\leq ...\leq \text{x}\left(\text{i}+\left({\text{j}}_{m}-1\right)\tau \right)$$

For *m* different numbers, there will be *j* = *m*! permutations. The vectors X(i) can be mapped to one of the *m*! permutations. Next, for the time series X(n), the probability of each permutation occurring (*p*) can be defined as:


(7) $$p_{j}=\frac{n_{j}}{\sum_{j=1}^{m!}n_{j}}$$

where *n*_*j*_ is the number of times the *j*th permutation is occurring.

The permutation entropy of the time series x(i) = [x (1), x (2), ‥., x (n)] is defined by:


(8) $$H_{x}(m)=\sum_{j=1}^{m!}p_{j}\text{ln}\;p_{j}$$

When the time series is random, *H*_*x*_*(m)* approaches its maximum value of ln(*m!*); when the time series is regular (non-random), *H*_*x*_*(m)* approaches zero. Finally, the corresponding normalized permutation entropy is:


(9) $$PE=\frac{H_{x}(m)}{\ln j}$$

The smallest value of *PE* is zero, which means the time series is very regular; and the largest value of *PE* is one, which means the time series is completely random.

##### 2.6.3.2. Fractal dimension (FD)

Earlier research has shown that EEG complexity analysis using FD may be employed successfully in a variety of clinical settings, implying that FD could be a good indicator of the efficacy of rTMS therapy (Lebiecka et al., 2018). The level of self-similarity of a time series is measured using an FD algorithm, which relates to how many times a pattern in the time series is repeated. FD was calculated using the Higuchi’s fractal dimension (HFD) technique (Ahmadlou et al., 2012; Bachmann et al., 2013; Čukić, 2020). The signal is represented by a sequence x = [x (1), x (2),‥., x(n)], where n is the total number of samples and x(i) indicates the *i*th sample of *x*. From a given sequence, *k* subsequences $X_{m}^{k}$ will be defined as:


(10) $$X_{m}^{k}:X\left(m\right),x\left(m+k\right),...,X\left(m+jk\right),m=1,2,...,k,$$

(11) $$j=\mathrm{int}\left(\frac{N-m}{k}\right)$$

where *m* is the initial time and *k* is the interval time.

The average length of each subsequence $X_{m}^{k}$ can be calculated according to:


(12) $$L_{m}^{k}=\frac{1}{k}\sum_{i,j}^{k}\left|X\left(m+ik\right)-X\left(m+\left(i-1\right)k\right)\left|\frac{N-1}{j}\right.\right.$$

where $\frac{N-1}{j}$ is a normalization factor. The total average length for scale *k*, *L(k)*, is computed as the average of the *k* values, for *m = 1, 2, ‥., k*, that is:


(13) $$L\left(k\right)=\frac{1}{k}\sum_{m=1}^{k}L_{m}^{k}$$

The calculation is repeated for *k* values ranging from 1 to k_max_. k_max_ is considered to be the number when the slope of the best line fitted to the diagram of *L(k)* vs. $\frac{1}{k}$ plotted in a log-log plane remains constant (k_max_ ≥2). The slope of this line is HFD of the time series *L*. The line is defined by the linear regression coefficient which is determined by the least squares method. k_max_ = 16 is shown to perform the best for this type of signal (Spasic et al., 2005; Lebiecka et al., 2018; Čukić et al., 2020).


(14) $$\text{Ln }L\left(k\right)∼HFD\text{ ln}\frac{1}{k}$$

##### 2.6.3.3. Lempel-Ziv Complexity (LZC)

The rate at which new patterns arise in time series is indicated by a nonlinear dynamic measure. The activities of adding or removing the patterns of the underlying system are examined when employing LZC (Aboy et al., 2006). The LZC method is based on coarse-graining, which converts the signal s(n) into a limited sequence of a few symbols (Li et al., 2008). Traditionally, the coarse-graining procedure turned the signal s(i) into a binary sequence x(i) = x (1), x (2),..., x(i). By comparing the signal values with a threshold, the original signal is converted into a binary (0, 1) sequence, with the median of the signal values chosen as the threshold.


(15) $$x\left(i\right)=\left\{\begin{array}{c} 0,\;if\;s\left(1\right)<T_{d}\\ 1,\;otherwise \end{array}\right.$$

Then by scanning the EEG sequences {x(i)} from the beginning, different patterns that appear in the signal are counted by *c(n)*. It has been proven that the upper limit of complexity measure *c(n)* with a median threshold is:


(16) $$\underset{n\to \infty }{\text{Lim}}c\left(n\right)=b\left(n\right)=\frac{n}{\log_{2}^{\left(n\right)}}$$

The complexity measure *c(n)* is normalized to *b(n)*, to make the measurement independent of the length of the data, and LZC will be obtained by:


(17) $$LZC=\frac{c\left(n\right)}{b\left(n\right)}$$

By transforming the signal into a finite sequence {x(n)}, c(n) will be the complexity of the sequence {x(n)} and will denote the number of distinct patterns in the sequence.

##### 2.6.3.4. Correlation dimension (CD)

Based on embedding theory and phase space reconstruction, the correlation dimension of a dynamic system is established. Considering the state points, x(1), x(2),‥.,x(n), new m dimensional vectors can be reconstructed by choosing a time delay *t* and embedding dimension *m*.


(18) $$\text{X}\left(\text{i}\right)=\left[\text{x}\left(\text{i}\right),\text{x}\left(\text{i+t}\right),...,\text{x}\left(\text{i+}\left(\text{m}-\text{1}\right)\text{t}\right)\right],\text{ i=1,2,}...\text{,n}-\left(\text{m}-\text{1}\right)\text{t}$$

The correlation dimension is obtained by:


(19) $$C\left(r,n\right)=\frac{2}{n\left(n-1\right)}\sum_{i=1}^{n}\sum_{j=i+1}^{n}\theta \left(r-\left|x_{i}-x_{j}\right|\right)$$

(20) $$\text{D}\left(\text{r, n}\right)=\underset{r\to 0}{\text{lim}}\frac{\text{ln C}\left(\text{r, n}\right)}{\text{ln r}}$$

where *θ* is the Heaviside step function which is defined as θ(x) = 0 for x < 0 and θ(x) = 1 for x > 0; *C(r, n)* is the correlation integral and D(r, n) is the correlation dimension. The procedure is repeated for increasing *m*.

#### 2.6.4. Bispectrum features

The spectral band power can show phase variations, but does not reveal interactions between the signal’s frequency components. Note that a signal’s “shape” is determined by its phase, and signals with different wave forms might have the same power spectrum (Acharya et al., 2015).

Bispectrum is a higher-order statistical signal processing technique that examines both the amplitude and the degree of phase coupling of a signal. In the context of EEG signal categorization, bispectrum has demonstrated promising results. The two-dimensional Fourier transform of a signal’s third order correlation is defined as the signal’s bispectrum *Bis(f1, f2)*. Bispectrum divides a signal’s skewness (third order moment) throughout all frequencies, characterizing the intensity of interaction between all frequency pairings in the target band.


(21) $$Bis\left(f_{1},f_{2}\right)=\underset{T\to \infty }{\text{lim}}\left(\frac{1}{T}\right)E\left[X\left(f_{1}+f_{2}\right)X^{*}\left(f_{1}\right)X^{*}\left(f_{2}\right)\right]$$

where *X(f)* is the Fourier transform of a time series *x(t)*, * is the complex conjugate, T represents time, and *E* denotes the expected value. Indeed, bispectrum indicates cross-correlation between frequency components in a two-dimensional frequency plot. The phase coupling information between the frequency components at f_1_, f_2_, and f_1_ + f_2_, can be extracted from Bis (f_1_, f_2_). The degree of phase coupling between frequencies components is obtained by the following normalized bispectrum:


(22) $$Bis_{norm}\left(f_{1},f_{2}\right)=\frac{Bis\left(f_{1},f_{2}\right)}{\sqrt{P\left(f_{1}\right)P\left(f_{2}\right)P\left(f_{1}+f_{2}\right)}}$$

The power spectrum is denoted by P(f), and the magnitude of the normalized bispectrum has a value between 0 and 1. The squared magnitude of the normalized bispectrum will be 1 if the Fourier components at the frequencies f1, f2, and f1+f2 are perfectly phase-coupled in every realization, and 0 if they are completely random.

To distinguish the distribution of the bispectral plots of distinct EEG data, quantitative characteristics must be defined. The bispectrum of a genuine signal is uniquely described in the triangle 0≤*f*_2_≤*f*_1_≤(f_1_+f_2_)≤1, assuming no bispectral aliasing. For characterization of the entire bispectrum, features are defined by integrating along straight lines going across the non-redundant region. Three features were collected from the bispectrum region in this investigation. These features are defined as follows.

**Figure 4:**
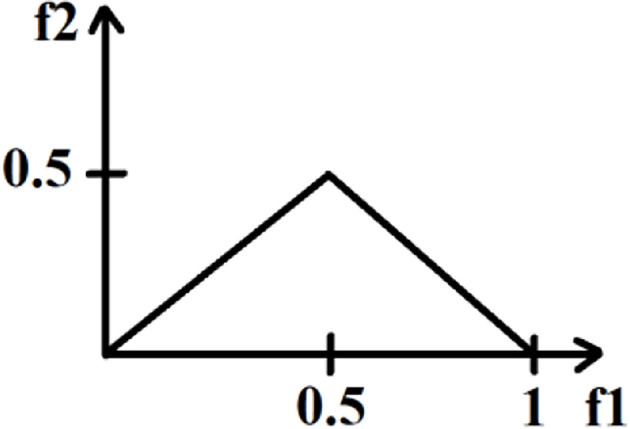
The non-redundant region of bispectrum plot.

Average of magnitude:


(23) $$M_{ave}=\frac{1}{L}\sum_{\Omega }\left|Bis\left(f_{1},f_{2}\right)\right|$$

where *L* is the total number of sample points in the bispectral density array and Ω is the triangle region shown in Figure 4.

Bispectral entropies:


(24) $$P_{1}=-\sum_{n}p_{n}\log p_{n}$$

(25) $$p_{n}=\frac{\left|Bis\left(f_{1},f_{2}\right)\right|}{\sum_{\Omega }\left|Bis\left(f_{1},f_{2}\right)\right|}$$

(26) $$p_{2}=-{\sum_{i}q_{i}\log q}_{i}$$

(27) $$q_{i}=\frac{{\left|Bis\left(f_{1},f_{2}\right)\right|}^{2}}{\sum_{\Omega }{\left|Bis\left(f_{1},f_{2}\right)\right|}^{2}}$$

where *P*_1_ and *P*_2_ are the normalized bispectral entropy and normalized bispectral squared entropy which are also between 0 and 1.

### 2.7. Feature selection

Before performing classification, we use feature selection for two main reasons: first, to eliminate the irrelevant features which can raise the complexity of classification and lower its accuracy, and second, to address the dimensionality problem. That said, after extracting EEG features, we applied a genetic algorithm (GA) to pick out the most discriminating features. GA is a group of computational models based on natural selection and genetics laws. In a high-dimensional space, it is considered as a stochastic strategy that outperforms deterministic optimization strategies. The GA begins with a population of individuals that represent a potential solution to a particular optimization issue and evolves over generations to a group of more optimal or fit individuals. Then, it replicates the offspring using basic genetic procedures such as selection, crossover, and mutation. A fitness value is assigned to each individual or community, and the fitness of the candidate individual is assessed. Finally, GA selects the best individuals from the existing population (Amoozegar et al., 2019).

### 2.8. Classification

We suggested a novel strategy to predict the rTMS treatment response based on classification analysis to separate responders from non-responders by the K-Nearest Neighbor (KNN), Support Vector Machine (SVM), and Multilayer Perceptron (MLP) classifiers. We ran our analysis on a different set of extracted features from the component time series. First, we assessed the capacity of the researched features to predict treatment response by applying classification to each measure individually. Then, in a second classification study, a mixture of features from each of the four categories (nonlinear, spectral, bispectral, and cordance) were utilized to classify R and NR. Finally, we assessed a combination of all features for classifying R and NR. For all analyses, the classification process detailed below was comparable. After feature extraction, the feature sets were separately standardized by the z-score normalization to minimize amplitude variations caused to differences in subjects and electrode placements. Afterwards, we employed GA to pick the most informative features.

In all analyses, the number of features was determined by a trial-and-error method to discover a value that maximizes accuracy while avoiding overfitting of the classifier. The square root of the total number of features in the related study was used to match this ideal number of features in our analysis, except for prefrontal cordance analysis, where due to the small number of features, we used all features.

A 10-fold cross-validation method was used to assess the classifiers’ performance. Except for two or three groups, the EEG data of 88 subjects were separated into 10 parts, with the number of signals in each part being equal. The classifier was trained using nine parts and the remaining part was used to test it. This process was performed 10 times, and the average and standard deviation of accuracy, sensitivity, and specificity were obtained.

#### 2.8.1. Multilayer perceptron (MLP)

A three-layer MLP with the error back propagation method and variable learning rate was used. For component time series (Ebrahimzadeh et al., 2011, 2018a,b, 2019d; Ebrahimzadeh and Pooyan, 2013; Nikravan et al., 2016), the input layer has the same number of nodes as the input vector length. The output layer, on the other hand, only has one node, which means that only two classes can be classified. The optimal number of neurons in the hidden layer was found by selecting and training all feasible combinations of the selected numbers of neurons in the hidden layer. It is worth noting that the entire training procedure was based only on the training data. We moved on to testing the network using the testing data after the training is completed. The network did not use the testing data while determining the best architecture to maximize the network’s generalization.

A linear transfer function and a sigmoid function were used in the output nodes and the hidden layer, respectively (Ebrahimzadeh et al., 2013). The network training continues until the error becomes less than 0.01, or 1,000 training iterations are completed (Ebrahimzadeh et al., 2014).

#### 2.8.2. K-Nearest neighbor (KNN)

After saving labeled feature vectors, this classifier calculates the shortest distance between saved and new feature vectors (Ebrahimzadeh et al., 2018a, c). The KNN algorithm is divided into three steps: (a) calculating distances between all previously classed samples; (b) selecting the K samples with the least distance values; and (c) approving new data. A new sample will be added (classified) to the largest cluster from the *K* selected samples. We looked at the values of *K* from 1 to 12 to compare with Hasanzadeh et al. (2019) and found that K = 7 delivers the best results. To make the tables easier to read, we used three *K* values (3, 7, and 12).

#### 2.8.3. Support vector machine (SVM)

SVM is a machine-learning technique that has proven to be effective in a variety of classification tasks. It focuses on the training examples near the edge of the class descriptors to find the optimal separating hyperplane (the plane with the largest margins) between the two classes of the training samples in the feature space. This method not only fits an ideal hyperplane, but also effectively uses less training samples, resulting in good classification accuracy with small training sets.

SVM is a well-known supervised learning model for classification and regression. The primary principle of SVM is to transfer the input data from the N-dimensional input space to the M-dimensional feature space M>N, where the data classes can be separated linearly (Ebrahimzadeh et al., 2018a). In other words, the SVM is a statistical learning theory-based extension of nonlinear models of the generalized portrait algorithm (Nikravan et al., 2016; Ebrahimzadeh et al., 2019d). The purpose of regression is to choose the best model from a group of models (known as estimating functions) in order to accurately anticipate future values. The estimation function for support vector regression is:


(28) $$\text{f}\left(\text{x}\right)=\left(\text{w.}\Phi \left(\text{x}\right)\right)+\text{b}$$

where *w⊂R_n_*, *b⊂R* and *Φ* is a nonlinear function that maps *x* into a higher dimensional space. *W* and *b* are the weight vector and bias, respectively. The weight vector (w) can be written as:


(29) $$w=\sum_{i=1}^{L}\left(\propto_{i}-\propto_{i}^{*}\right)$$

By substituting Equation (29) into Equation (28), the generic equation can be rewritten as:


(30) $$f\left(x\right)=\sum_{i=1}^{L}\left(\propto_{i}-\propto_{i}^{*}\right)\left(\Phi \left({\text{x}}_{\text{i}}\right).\Phi \left(\text{x}\right)\right)+\text{b.}$$

(31) $$f\left(x\right)=\sum_{i=1}^{l}\left(\propto_{i}-\propto_{i}^{*}\right)k\left(xi.x\right)+b$$

where the function *k (xi . x)* = *(Φ(x_i_). Φ(x))* is known as the kernel function and *∝* = *(∝ 1, ∝ 2, ..., ∝ l)* is the vector of non-negative Lagrange multipliers.

The choice of kernel functions and kernel parameters depends mainly on the application. Among the useful kernel functions are radial basis functions (RBFs) and polynomial kernel functions:


(32) $$\left\{\frac{-{\left|x-xi\right|}^{2}}{2\sigma^{2}}\right\}$$

(33) $${\left[\left({\text{x*x}}_{\text{i}}\right)\text{+1}\right]}^{\text{d}}$$

where *σ* and *d* are kernel width and order, respectively, which were experimentally defined to achieve the best classification result. In this work, RBFs and polynomial kernel functions were used with different sigma values (σ = 0.8, 1, 1.2) and orders (d = 1, 2, 3).

### 2.9. Statistical comparison

Statistical testing seeks to understand how a system works, whereas machine learning approaches seek to anticipate future behavior (unobserved consequence; Bzdok et al., 2018). Therefore, they can be used in conjunction with the current investigation to provide a more thorough assessment of the studied features’ potential to detect R and NR responses to rTMS treatment. We also ran a statistical test to compare the results of our data to those of earlier studies that used statistical analysis. Since some of the data did not have a normal distribution, we used analysis of variance (ANOVA) to compare every feature between the two groups.

### 2.10. Evaluation

Accuracy (AC), Sensitivity (SN), Specificity (SP), and Precision (P) are used to assess the proposed method’s capacity to predict response to rTMS treatment. TP stands for true positives (correctly predicted R), TN for true negatives (correctly predicted NR), FN for false negatives (incorrectly predicted NR), and FP for false positives (incorrectly predicted R).

Accuracy (AC): the ratio of correct predictions to the total predictions


(34) $$\text{AC = }\frac{\text{TP + TN}}{\text{TP + TN+ FN+ FP}}$$

Sensitivity (SN): the ratio of true positives to the total positives


(35) $$\text{SN = }\frac{\text{TP}}{\text{FN + TP}}$$

Specificity (SP): the ratio of true negatives to the total negatives


(36) $$\text{SP = }\frac{\text{TN}}{\text{TN + FP}}$$

Precision (P): the ratio of predicted positive cases that were correct


(37) $$\text{P = }\frac{\text{TP}}{\text{FP + TP}}$$

## 3. Results

**Figure 5:**
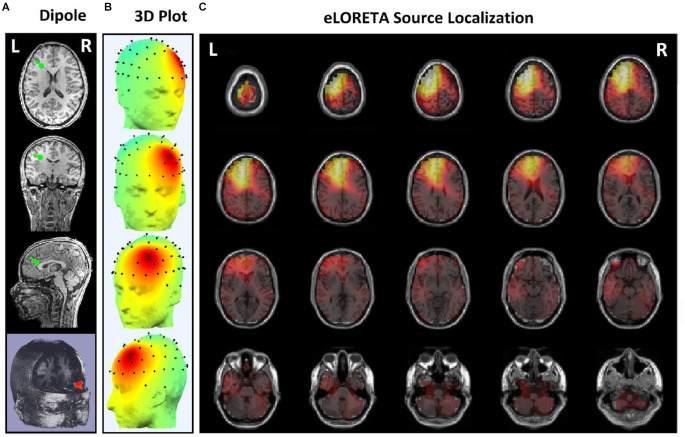
Source localization of rTMS stimulation foci in left prefrontal cortex (L-DLPFC) for a responder subject. **(A)** Dipole coordinates of the identified focus in left side on a default MRI. **(B)** Position of foci identified by ICA on the surface of the skull. **(C)** Source localization in right DLPFC by eLORETA.

**Figure 6:**
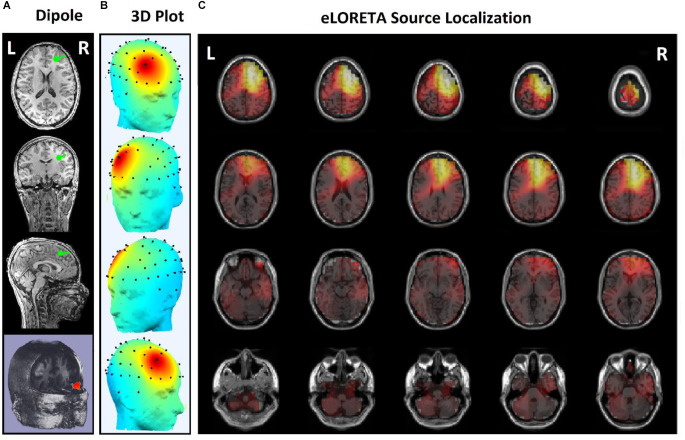
Source localization of rTMS stimulation foci in right prefrontal cortex (R-DLPFC) for a non-responder subject. **(A)** Dipole coordinates of the identified focus in right side on a default MRI. **(B)** Position of the foci identified by ICA on the surface of the skull. **(C)** Source localization in right DLPFC by eLORETA.

**Figure 7:**
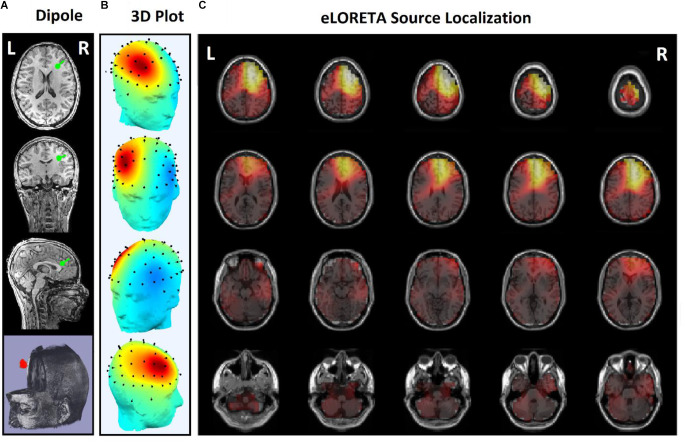
Source localization of the rTMS stimulation foci in right prefrontal cortex (R-DLPFC) for a responder subject. **(A)** Dipole coordinates of the identified focus in right side on a default MRI. **(B)** Position of the foci identified by ICA on the surface of the skull. **(C)** Source localization in right DLPFC by eLORETA.

**Figure 8:**
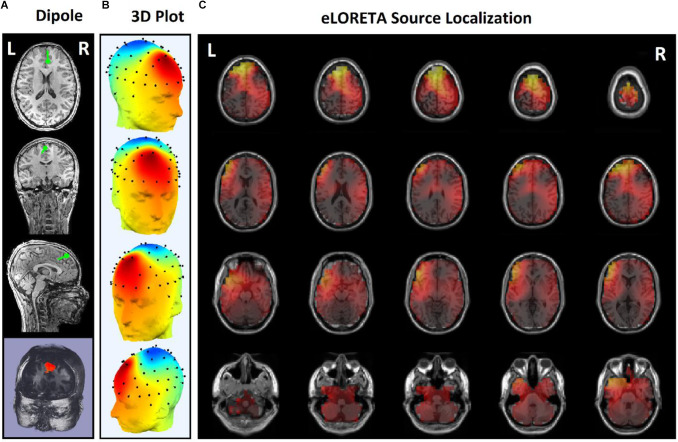
Source localization of the rTMS stimulation foci in center of frontal for a responder subject. **(A)** Dipole coordinates of the identified focus in the center of frontal region on a default MRI. **(B)** Position of the foci identified by ICA on the surface of the skull. **(C)** Source localization in right DLPFC by eLORETA.

Since the effects of stimulation on inactive neural networks in the frontal and pre-frontal areas as brain sources are always greater than EEG channels, we first identified the rTMS-stimulated foci in the mentioned areas and then extracted nonlinear features from the desired foci. In order to identify the relative foci, first the location of the focus in the standard MNI is visually determined and after confirming the dipole coordinates of the representative of that focus in the frontal region, the desired component is determined as the excitable focus. The obtained results indicate the presence of foci in the L-DLPFC (Figure 5), R-DLPFC (Figures 6, 7), and center of DLPFC (Figure 8) each of which is confirmed by the dipole coordinates and eLORETA results.

Figure 5 shows the position of a focus identified by the ICA algorithm, which is also confirmed by eLORETA algorithm. The focus shown on the left side of DLPFC belongs to a responder subject.

Figures 5 and 6 are the identified foci on the right side of the prefrontal region that belong to a NR subject and a R subject, respectively. It is noteworthy that although the NR subject has a recognizable focus in the mentioned area, the extracted features reveal a significant difference between the NR and R subjects in terms of prediction of the rTMS treatment which will be explained further.

In some subjects undergoing bilateral treatment, the main focus is localized in the center of the frontal region. These patients often have symptoms of depression as well as insomnia and anxiety (Figure 7).

### 3.1. Analytical results

We extracted 23 features from each component time series: four nonlinear features, including PE, LZC, FD, and CD, four power features, which contain delta, theta, alpha, beta bands, and 12 bispectrum features, i.e., BispSL, Bisp2M, and BispEn in all bands, and three cordance features, i.e., IC1CORD-T, IC2CORD-T, and IC3CORD-T features in theta band. Then, by applying the GA feature selection, 12 features were selected. Our study shows that these selected features from the relevant components can be used as suitable markers for prediction of rTMS treatment response. In other words, the components identified in DLPFC can be a more suitable platform and tool for extracting effective features.

Table 2 depicts the efficiency of the 23 selected features in predicting rTMS treatment response in MDD, which was evaluated by analysis of variance (ANOVA) on the learning set. According to this table, all of the bispectrum, cordance, and non-linear features had acceptable discriminating power, as implied by *P*-Value. The results of ANOVA test in spectrum features also showed that T, A, and B power differ significantly between R and NR, while due to the high *P*-Values of frequency peaks in the Delta band, these features cannot distinguish the two groups with statistical significance. Therefore, we used 22 features with statistically significant discrimination, excluding the frequency peaks in Delta band from the aforementioned features to form the feature vector.

**Table 2:** Results of the ANOVA test.

**Extracted features from components**	***P*-Value**
Nonlinear (PE, LZC, FD, CD)	0 ≤ 0.0001
Power (Theta, Alpha, Beta)	0 ≤ 0.0001
Power (Delta)	0.04
Bispectrum (BispSL, Bisp2M, and BispEn in all bands)	0 ≤ 0.0001
Cordance (IC1, IC2 and IC3)	0 ≤ 0.0001

In this study, our main focus is to use the genetic algorithm as a more advanced method of feature selection. After an initial evaluation of features, their optimal combination, consisting of 12 features, was selected by GA. The SVM classifier parameters and kernel width (σ) must be chosen with attention and caution to optimize the learning cost and prediction performance. To do so, we compared the classifier’s performance by evaluating the error function using an independent validation set and selecting the classifier with the minimum error relative to the validation set. Because this technique can cause overfitting in the validation set, the test dataset was used to confirm the performance of the chosen classifier. In other words, based on a 10-fold cross-validation, the data were divided into 10 equal parts and in each stage, 10% of the data were used as the test dataset and the rest of the data as the training dataset. In fact, nine of the observations were selected as test data and 79 as train data, and this process was repeated 10 times. The optimum values of the parameters, chosen when the error on the validation dataset reached a minimum, were 0.7 for σ and 3 for order *(d)*.

Table 3 illustrates the performance of the two classifiers with variable parameters, i.e., SVM and KNN. The features were used as input of GA to reduce their number and improve the proposed algorithm’s performance. For classification, we utilized SVM and KNN. We assessed the performance of classifiers with various kernels and Ks, such as polynomials of order 1, 2, and 3, and RBFs with various sigma values (σ = 0.7, 1, 1.3) for the SVM classifier. We tested values of *k* from 1–13 and found that *k* = 7 in the KNN classifier achieved the best results. To reduce the complexity of Table 3, we display four k-values (3, 7, 10, and 13). It is highlighted that the input of the classifiers is the components features extracted by GA.

**Table 3:** Performance of the KNN and SVM classifiers with different parameters using the combined feature vector.

**Classifier**	**Sensitivity**	**Specificity**	**Accuracy**
SVM-Poly (d = 1)	81.37%	97.12%	93.52%
SVM-Poly (d = 2)	83.24%	97.40%	92.24%
SVM-Poly (d = 3)	83.63%	97.41%	93.72%
SVM-RBF (σ = 0.7)	82.61%	98.77%	93.21%
SVM-RBF (σ = 1.0)	81.17%	98.87%	93.19%
SVM-RBF (σ = 1.3)	81.44%	97.63%	92.75%
KNN (K = 3)	77.26%	96.37%	92.37%
KNN (K = 7)	80.36%	97.12%	93.18%
KNN (K = 10)	78.12%	96.22%	89.17%
KNN (K = 13)	76.44%	94.22%	87.64%

Four measures based on Equations (34) to (37) were used to evaluate the performance of the proposed approach. If for example, a responder (R) is correctly classified as R, it is a TP. On the other hand, if an NR is classified as NR, it is a TN. Any NR that is wrongly classified as an R will result in an FP, whereas any R that is mistakenly classified as an NR will result in a FN. Table 4 summarizes the results of the test data classification for each class.

**Table 4:** Classification results by SVM, KNN, and MLP, using combined feature vectors.

	**TP**	**FP**	**FN**	**TN**
SVM	44	3	2	39
KNN	41	6	5	36
MLP	42	5	4	37

The obtained sensitivity, specificity, precision, and accuracy of the proposed method are shown in Table 5. Note that the SVM classifier outperformed the other classifiers and achieved 94.31% accuracy, 95.65% sensitivity, 92.85% specificity, and 93.61% precision, respectively.

**Table 5:** Classifier performance in percentage, for 10-fold cross validation and combined feature vector.

**Classifier**	**Accuracy (%)**	**Sensitivity (%)**	**Specificity (%)**	**Precision (%)**
SVM	94.31	95.65	92.85	93.61
KNN	87.50	89.13	85.71	87.23
MLP	89.77	91.30	88.09	89.36

We applied feature sets extracted from components time-series to classifiers in the following forms to evaluate the capability of the selected features in the prediction of response to rTMS treatment: each feature independently, the combination of features in each group of nonlinear, spectral, bispectral, and cordance features. Our proposed classification approach was evaluated using the following criteria: classification accuracy, specificity, sensitivity, and precision. Table 6 shows these criteria in relation to each measure which were calculated based on the three components with the highest average λ (weight of extracted independent components) in the frontal area and reduced to 12 features by GA. The evaluation of classification by the combination of studied features is also given in Table 6. As can be seen, combinational features are more capable than the individual features which is why they have been used in this study as the input to predict the rTMS treatment response.

**Table 6:** Accuracy of SVM, KNN, and MLP classifiers with the selected features (individually and in combination) for classifying NR and R.

**Features**	**SVM**	**KNN**	**MLP**
Non-Linear	84.91%	76.82%	78.24%
Power	91.82%	78.13%	80.66%
Bispectrum	89.23%	81.67%	82.15%
Cordance	76.18%	82.73%	71.63
Combination	94.31%	87.50%	89.77%

## 4. Discussion

Identification of biomarkers for predicting the therapeutic outcome of antidepressant treatment by rTMS is the main goal of the current research as it can transform the lengthy process of finding the right treatment for patients with MDD. We have explored the potential of pretreatment cortex activity and extracted features from components as a putative biomarker of treatment response.

Given that EEG channels do not always represent the foci stimulated by rTMS, in this study, we focused on the extraction of affected foci in the frontal region. The components in the frontal lobe indicate the neural behavior of networks that are activated by electromagnetic stimulation and point to the sources activated during treatment. It seems that one of the factors that can elucidate the improved results of this article, compared to those of previous studies, is the analysis of temporal behavior of the relevant sources rather than the EEG channels. As the classification results illustrated, our proposed method led to high classification accuracy. Table 7 compares our results with those of the previous studies that applied machine learning techniques for prediction of MDD treatment response.

**Table 7:** Comparison of classification results of studies that applied machine learning techniques for prediction of MDD treatment response with those of the current study.

**Features**	**Accuracy (%)**	**Specificity (%)**	**Sensitivity (%)**
Band Power (Metin et al., 2020)	80.0	-	-
Power Spectral, eLORETA, multiscale-entropy-based, (Zhdanov et al., 2020)	79.2*	91.0	67.3
MADRS, PSD, iAPF, theta Cordance, wPLI (Bailey et al., 2019)	86.60	89.0	84.0
MADRS, eLORETA, Theta Cordance (Jaworska et al., 2019)	87.6	90.3	83.2
Theta frequency band (Erguzel et al., 2015)	89.1	-	94.4
PSD, Alpha asymmetry (Al-Kaysi et al., 2017)	76 (Mood) 92 (Cognition)	-	-
Power Spectral, , cordance, asymmetry (Cao et al., 2018b)	81.3	82.1	91.9
Wavelet (Mumtaz et al., 2017)	87.5	95	80
PSD, PSD ratio, Coherence, Mutual Information (Khodayari-Rostamabad et al., 2013)	87.9	80.9	94.9
MADRS, Working Memory accuracy, PSD, wPLI (Bailey et al., 2018)	91.0	92.0	91.0
Non-Linear, power, Bispectrum, cordance (Hasanzadeh et al., 2019)	91.3	91.3	91.3
Time-Frequency domain analysis (Ebrahimzadeh et al., 2021a)	82.43	75.0	86.0
Our proposed method based on components analysis	94.31	92.85	95.65

*Only the baseline EEG data, MADRS, Montgomery and Asberg Depression Rating Scale; PSD, power spectral density; wPLI, weighted phase lag index.

In an attempt to formulate a method for prediction of rTMS clinical effects, we obtained various features from pretreatment resting-state EEG of patients with MDD who had undergone the rTMS procedure, including 34 non-responders and 34 responders. While much of the literature is dedicated to the power of alpha and theta frequency bands (Bailey et al., 2018, 2019), our data analysis results suggest that beta power, when derived from the relevant component time-series, can make for a prognostic biomarker of more significant potential. Similarly, a number of studies such as Lieber and Prichep (1988) and Knott et al. (2000) show that a high rate of beta activity is associated with the depression level. It can then be concluded that the high beta power is linked to lower levels of treatment responsiveness. As for other frequency bands, although theta and alpha bands produced a rather high classification accuracy, only alpha and delta power could be used to statistically differentiate between the two groups (Olejarczyk et al., 2020). Our results confirm that responders exhibit a greater pretreatment alpha power compared to non-responders, which has also been observed in other studies including (Ulrich et al., 1984; Suffin and Emory, 1995; Knott et al., 2000; Bruder et al., 2001, 2008; Lebiecka et al., 2018). Bruder et al. (2008) elucidated that this increased alpha power could point to the correspondence between low arousal and low serotonergic activity. They showed that the fact that 5-HT mediates arousal and serotonergic activity might be suggestive of the activity of the mesencephalic raphe nuclei and cortical afferents (Bruder et al., 2008).

Heller et al. (1995) linked depression to dysfunction of temporoparietal mechanisms which may mediate emotional arousal. They found that delta power was lower in rTMS responders than in non-responders, which was also highlighted in other studies such as Knott et al. (1996) and Knott et al. (2000). Our results depict the significant differences in frontal delta power of responders and non-responders. In comparison, however, the power of theta frequency band has been reported to be of less relevance since both responders (Woźniak-Kwaśniewska et al., 2015) and non-responders (Arns et al., 2012) seemed to have high theta power. We also detected no major differences between the two groups with relation to the theta power. This was also the case in a previous study (Cook et al., 1999). Furthermore, we found the nonlinear features to be of great potential in predicting rTMS treatment response, while they have been relatively overlooked in the literature. The correlation of dimension (CD), for example, was considerably lower in responders, which produced a high classification accuracy of 87%.

We also investigated the predictive abilities of frontal and prefrontal theta cordance which led to classification accuracy of 80.4% and 78.3%, respectively. Our statistical analysis shows that these measures fail to differentiate between the two groups. This is in line with the results of other studies such as Arns et al. (2012) and Bailey et al. (2019).

To evaluate the outcomes, we applied different classifiers and achieved the accuracy of classification using the determined features both individually and in combination (Table 6). Then, we drew a comparison between the obtained results and those of previous works such as (Hasanzadeh et al., 2019; Table 7). As shown, the proposed method has outperformed the previous ones, except for the result of the cognition output in the study of Al-Kaysi et al. (2017), where they aimed to predict mood and cognition output separately. To this end, they designated 0 or 1 values to mood and cognition based on whether their scores from the middle of treatment had decreased or increased compared to the baseline scores. They then used machine learning techniques to predict theses values, also known as the outputs, based on the EEG features. It should be highlighted that after the treatment, the cognition output exhibited alterations in the Symbol Digit Modalities Test (SDMT). The results of cognition output are irrelevant to depression therapy prediction because SDMT is not a depression severity rating scale. The mood prediction, on the other hand, highlighted changes in MADRS with a comparatively low accuracy of 76%. A similar study (Bailey et al., 2018) employed both mood and EEG measures and achieved higher accuracy.

The mood measures are generally subject to expert rating and therefore can be different from one to another. Since the proposed method is solely based on the EEG activity, we believe it is more appropriate than that of the mentioned study. In addition, Bailey et al. (2018) separated the two groups of responders and non-responders using two sessions of EEG, one prior to the treatment and one after a week of treatment, whereas we have used only one session of pretreatment EEG and obtained high classification results (accuracy = 91.3%, specificity = 91.3%, sensitivity = 91.3%). The fact that only one session of EEG recording is required, brings to attention the efficiency of the proposed method. It also lifts the financial and mental burden of undergoing a week-long, possibly ineffective treatment.

The major novel aspect of this study was applying predictive analytics and machine learning on the component time-series extracted from scalp EEG to interpret and summarize the neural activity in DLPFC. We were able to identify outstanding features from a big amount of data using machine learning to recognize outcome predictors that were previously undetectable. This capability is greatly enhanced by using time series of foci identified as stimulated neural networks. In fact, the proposed method indicates the high ability to predict the response to treatment based on neuronal activity.

We obtained these results using EEG and machine learning in a standard clinical setting of rTMS therapy for MDD, indicating that these methods are useful tools for studying cortical networks and possibly guiding TMS treatment. We were able to restrict the data’s intrinsic complexity and choose electrophysiological variables relevant to therapy-induced alterations and response prediction using the algorithms we used. This method shows potential in constructing treatment regimens, devices, and measurements to enable screening and personalizing treatment viable in the office setting, given the price and accessibility of EEG and the application of data-driven approaches.

This study aimed to evaluate features of extracted components from EEG as potential predictors of MDD treatment responses in patients who received excitatory rTMS to L-DLPFC. The results denote that EEG decomposition components embody different energies in different patients which can be used to separate the responders from non-responders. We show that performing non-linear analyses on the time-series of the EEG components can lead to more promising results and reliable predictors of rTMS treatment response, in comparison with the classical linear methods. Furthermore, we applied MLP, KNN, and SVM classifiers to different features extracted from pretreatment EEG, including nonlinear, power spectrum, bispectrum, and cordance extracted from ICs, and also a combination of them. Component’s power of beta and power of all frequency bands yield classification accuracy of 94.3%, which explains that component power, particularly in beta band, is a valid biomarker of treatment response. Correlation dimension and some of the features based on bispectrum amplitude also have significant predictive abilities. We believe that the appropriate results of the proposed method are suggestive of its potential for clinical applications.

### 4.1. Limitations and future research directions

The lack of a sham condition is one of the work’s limitations. Previous work points to changes in cortical networks during placebo responses (Cook et al., 2002; Hunter et al., 2006; Benedetti et al., 2011). Furthermore, brain connectivity on functional imaging may predict placebo responses (Tétreault et al., 2016), which emphasizes the importance of future sham-controlled studies. Although the size of our samples is larger than other studies, it is still relatively small compared to the extracted features. Overfitting, in which the algorithm learns tendencies in the data that are not generalizable to new samples, is a common concern with such studies. We tried to address this by employing regularization (reducing model complexity), cross-validation (training and testing the model on different data), and shuffling (demonstrating that the suggested approach does not learn random patterns in the data). The other limitation that this sample size creates is that the variables setting apart the responders from non-responders can only be identified based on their response to rTMS treatment in general. Therefore, replication with a larger sample size is needed to confirm the validity of our findings.

Another concern is that it is possible for the results to have been affected by unpredictable sampling bias. For one thing, most of the participants were not quite sure about the number of their prior depressive episodes. More detailed clinical features such as the current medication dosage were also not recorded. In addition, it is relatively likely that we have dismissed EEG measures with high predictive potential. The sample size also did not allow for a separate analysis of left and right, and bilateral rTMS treatment response in the randomization phase of the study after the initial 3 weeks of left-sided treatment (which was consistent across all participants). Although, to our knowledge, previous research has not yet detected such differences (Fitzgerald et al., 2018), we still believe that deciding if and to what extent left or right-sided rTMS treatment is effective for a patient seems to be a priority for future research work.

Notwithstanding these limitations, we discovered reliable and therapeutically relevant results. To maximize the benefits from this research, future work can be focused on the following:

(1) Independent replication in a larger sample to confirm the predictive ability of the features chosen in this study, or the most predictive features from the literature. (2) Once the predictive ability of our features is proven to be effective in large samples, the focus then should be laid on simplifying and manualizing the calculational procedures to enable a more practical clinical application. An improved machine learning algorithm (perhaps using deep learning for more sophisticated prediction in larger datasets) could then use anonymous data from patients across clinics for better prediction accuracy and generalizability. (3) Research should be conducted to find out whether the outcomes for the patients categorized as non-responders would be worse if they undergo failed treatment as opposed to not undergoing the treatment altogether. If it is established that non-responders show increased depression severity after undergoing unsuccessful treatment, there will be a strong case that the prediction of non-response should result in referrals to an alternative treatment (rather than undergoing rTMS treatment). However, if outcomes remain relatively the same for participants undergoing an unsuccessful treatment, the treatment could still benefit the patient, because even if response prediction outcomes are accurate, there is still a small chance patients will respond in reality (Bailey et al., 2018). (4) Regardless of the answer to point 3, there is still an obvious need for alternative treatment methods to be developed for non-responders. With regard to brain stimulation, this could entail predicting whether left or right-sided treatment is most suited for particular individuals, and applying rTMS to the medial prefrontal cortex instead of the DLPFC (Dunlop et al., 2017). (5) Investigating the statistical effect of treatment heterogeneity as a paradigm on their classification matrices. Ideally, the type of treatment should be entered as a covariant in the analyses to determine if it has a significant effect on classification.

## 5. Conclusion

The aim of this study was to evaluate the ability of components extracted from EEG to predict response of rTMS treatment in MDD patients. As expected, the features extracted from the foci of the frontal region had more capability and capacity to differentiate between the two groups (R and NR) than the EEG channels. We have also shown through the use of an advanced machine learning method that nonlinear and frequency features of the components can predict rTMS treatment response. The obtained results show that the proposed method is more capable than other similar methods. Machine learning successfully predicted lack of response to rTMS with high specificity and identified pre- and post-rTMS status using EEG coherence. This approach may provide mechanistic insights and may also become a clinically useful screening tool for rTMS candidates. In future studies, we intend to obtain defined features from other sources decomposed from EEG to improve the classification accuracy. In addition, examining sources identified in other areas of the brain can also increase the yield of studies in the field of rTMS treatment. Further work can also be done on the application of the proposed method to other neurological diseases such as migraine and obsessive-compulsive disorder.

## Data availability statement

The raw data supporting the conclusions of this article will be made available by the authors, without undue reservation.

## Ethics statement

The studies involving human participants were reviewed and approved by the local ethics committee of the Iran University of Medical Sciences. The patients/participants provided their written informed consent to participate in this study. Written informed consent was obtained from the individual(s) for the publication of any identifiable images or data included in this article.

## Author contributions

EE, MA, and HS-Z conceived the presented idea. EE developed the theory and performed the computations. Material preparation, data collection, and analysis were performed by EE, FF, MS, and MA. The first draft of the manuscript was written by EE, FF, and MS and all authors commented on previous versions of the manuscript. LR and HS-Z verified the analytical methods. The visualization and validation were done by EE. All authors provided critical feedback and helped shape the research, analysis, and manuscript. All authors read and approved the final manuscript. HS-Z supervised the project.

## Footnotes

^1^ http://www.sciencebeam.com/
